# Radioimmunotherapy-based conditioning regimen prior to autologous stem cell transplantation in non-Hodgkin lymphoma

**DOI:** 10.2217/ijh-2017-0025

**Published:** 2018-06-13

**Authors:** Mahsa Eskian, MirHojjat Khorasanizadeh, Alessandro Isidori, Nima Rezaei

**Affiliations:** 1 Research Center for Immunodeficiencies, Children's Medical Center, Tehran University of Medical Sciences, Tehran, 14194, Iran; 2 Network of Immunity in Infection, Malignancy & Autoimmunity (NIIMA), Universal Scientific Education & Research Network (USERN), Tehran, Iran; 3 Haematology & Haematopoietic Stem Cell Transplant Center, AORMN Marche Nord Hospital, Via Lombroso 1, 61122 Pesaro, Italy; 4 Department of Immunology, School of Medicine, Tehran University of Medical Sciences, Tehran, Iran; 5 Cancer Immunology Project (CIP), Universal Scientific Education & Research Network (USERN), Tehran, Iran

**Keywords:** autologous stem cell transplantation, conditioning, non-Hodgkin lymphoma, radioimmunotherapy, toxicity

## Abstract

Non-Hodgkin lymphoma (NHL) is the most common hematologic malignancy and the sixth cause of death from cancer in the USA. Autologous stem cell transplantation (ASCT) is a potentially curative therapeutic option for many NHL patients. Choosing the most effective conditioning regimen prior to ASCT can lead to longer survival in these patients, and, as in many cases of high risk NHL, the only potentially curative option is stem cell transplantation. Radioimmunotherapy (RIT) is based on using radiolabeled monoclonal antibodies against tumoral antigens. Since lymphoma cells are sensitive to radiation, RIT has become a potential approach in treating NHL. In this review, we have discussed the efficacy and safety of RIT as an alternative conditioning regimen prior to ASCT.

**Practice points**

- For high-risk and chemorefractory non-Hodgkin lymphoma patients there is a need for a novel conditioning regimen administered prior to autologous stem cell transplantation.
- Adding standard dose of radioimmunotherapy (RIT) agents to conventional chemotherapy conditioning regimens significantly improves rate and duration of response.
- For high risk and elderly non-Hodgkin lymphoma patients adding high-dose RIT to conditioning regimen could provide more promising and durable response.
- The most common toxicity of RIT as conditioning regimen is hematologic toxicity, which is reversible.
- The rate of nonhematologic toxicities, secondary malignancies, and mortality are not significantly different in RIT-containing conditioning regimens in comparison with conventional conditioning regimens.
- Time to engraftment is not prolonged by adding RIT to conditioning regimen prior to autologous stem cell transplantation.

Non-Hodgkin lymphomas (NHLs) are malignant neoplasms of B, T, and natural killer cells [1]. NHL is the most common hematologic malignancy and the sixth cause of death from cancer in the USA [2]. High dose therapy (HDT) followed by autologous stem cell transplantation (ASCT) is a potentially curative therapeutic alternative for resistant or relapsed NHL patients [3]. Even for many cases of high risk NHL, the only potentially curative option is stem cell transplantation [4]. Success of hematopoietic stem cell transplantation (HSCT) in treating NHL has increased in recent years [5], and promoting the conditioning regimen prior to  HSCT can further increase this success [6]. However, total body irradiation (TBI) as a conventional conditioning regimen is associated with life threatening adverse events such as cardiac toxicities, infections, and interstitial lung disease with high mortality rate [7–9]. Moreover, some NHL patients could not benefit from the chemotherapy conditioning regimen due to the chemorefractory conditions of their tumor [10–12]. Therefore, in parallel to novel chemotherapeutic agents, the incorporation of radioimmunotherapy (RIT) in HDT prior to ASCT in B-cell aggressive lymphomas was tested in several clinical trials. RIT is based on targeting tumoral antigens with radiolabeled monoclonal antibodies. Two radioimmunoconjugates including Bexxar^®^ (^131^I-tositumumab) and Zevalin^®^ (^90^Y-ibritumomab tiuxetan) have been approved by US FDA; although only ^90^Y-ibritumumab is available in the USA because of marketing reasons [13]. RIT's ability in eliminating residual disease [14–16], and lymphoma cells’ sensitivity to radiation [17], can make it a new promising alternative conditioning regimen prior to ASCT. In this paper the role of RIT as a novel conditioning regimen prior to ASCT has been reviewed.

## Efficacy

There are several studies which have reported favorable results of using RIT in combination with stem cell transplantation [18,19]. Using a radiolabeled antibody as a part of the pretransplantation conditioning is a promising approach to increase the feasibility and efficacy of ASCT [8,20].

### Standard dose of RIT

Standard dose of RIT in combination with BEAM (carmustine, etoposide, cytarabine, and melphalan) as the conditioning regimen for ASCT has been investigated in several studies. In the study by Mei and his colleagues, 90Y-ibritumomab tiuxetan in combination with BEAM prior to ASCT in 63 patients with transformed NHL showed impressive results, with 2-year overall survival (OS) approaching 90%. A total of 30 (48%) patients underwent ASCT at their first complete remission which could be a possible explanation for this promising result [21]. In another study standard dose of 131I-tositumomab plus high dose BEAM followed by ASCT in 40 relapsed or refractory  diffused large B-cell lymphoma (DLBCL) patients resulted in a promising result of 80% overall response rate (ORR) with 78% complete response (CR). With median follow-up of 6 years, the median 5 years OS and progression-free survival (PFS) were 72 and 70%, respectively [22]. In another study, 23 patients with chemorefractory or multiply relapsed NHL were treated with RIT and BEAM followed by ASCT and reached a total CR rate of 57% and ORR of 65%. After a follow-up of 38 months, the event-free survival (EFS) was 39%, which is higher than studies in similar high risk lymphoma patients who underwent conventional HDT prior to ASCT, with 10–20% survival rates at a comparable time [23,24]. In a randomized study in 43 NHL patients, Shimoni *et al*. compared 90Y-ibritumomab tiuxetan in combination with BEAM versus BEAM alone prior to ASCT. They showed that in multivariate analysis that BEAM regimen was a significantly adverse prognostic factor for PFS (p = 0.03) and OS (p = 0.01) in comparison with RIT and BEAM combination [25].

Recently, ten studies enrolling a total of 328 patients with DLBCL treated with 90Y-ibritumomab tiuxetan and BEAM followed by ASCT were pooled together in a meta-analysis. The results of the meta-analysis showed that patients with RIT-BEAM had higher rates of OS and PFS at 2 years (p < 0.01 and p < 0.05, respectively) than those who received BEAM alone. For Z-BEAM regimen the 2-year OS and PFS were 84.5 (n = 328) and 67.2% (n = 285), respectively [26]. These favorable results of adding RIT to standard BEAM may be due to the ability of RIT in eliminating the residual disease, as there are several studies showing the ability of RIT consolidation to convert partial response (PR) to CR following conventional induction therapies [27,28].

Based on our knowledge there are few studies which have investigated the combination of standard dose of RIT with other chemotherapy regimens other than BEAM as conditioning regimen. In a study of 90Y-ibritumomab plus busulfan, cyclophosphamide and etoposide (BU/CY/E) followed by ASCD, in relapsed or refractory NHL patients, in a median follow-up of 29.4 months the estimated 3-year OS and EFS were 52.5 and 26.3%, respectively [29]. In another study Jo *et al*. compared the efficiency of 90Y-ibritumomab tiuxetan combined with BuCyE with BuCyE alone as conditioning regimen prior to ASCT in 38 relapsed or refractory NHL patients. In a median follow-up of 30.4 months there were no significant difference in EFS and OS observed between the two groups [30].

Regarding comparison of RIT with rituximab, Vose *et al*. compared rituximab-BEAM with 131I-tositumomab-BEAM prior to ASCT in 234 chemotherapy-sensitive relapsed DLBCL patients. The 2 year PFS rates were 48.6% for rituximab group and 47.9% for RIT group (p = 0.9) and 2 year OS rates were 65.6% for rituximab and 61% for RIT group (p = 0.3) [31].

Based on aforementioned studies it seems that adding RIT to conventional conditioning regimens significantly enhances the efficacy of the conditioning regimen prior to ASCT. However, RIT has no superiority to rituximab in this area although more studies are needed. Regarding different chemotherapy agents, more studies are needed to clarify the best one for combination with RIT as a conditioning regimen.

### High-dose RIT

Myeloablative dose of RIT, alone or in combination with other treatments, as a preparative regimen prior to ASCT in patients with high-risk NHL was investigated in some studies. As a whole, prolonged remissions were achieved [32–34], even in elderly patients [35]. Winter *et al*. showed that using myeloablative doses of RIT as a conditioning regimen can make promising results even in patients who are not eligible for transplantation [36].

Gopal *et al*. investigated the high dose 131-I tositumomab followed by high dose etoposide and cyclophosphamide in 11 relapsed mantel cell lymphoma (MCL) patients followed by ASCT. The interesting results of 91% CR and 100% ORR with 3-year OS of 93% were achieved [37]. In another study Cassaday *et al*. compared adding high-dose 131I-tositumomab RIT to conventional conditioning with conventional HDT prior to ASCT in 162 persistent MCL patients. A total of 61 patients received RIT-based HDT and 101 patients received conventional conditioning regimen including TBI with or without chemotherapy. In multivariate analysis, high-dose RIT conditioning was significantly less likely to cause treatment failure compared with conventional HDT conditioning (p = 0.001) [38]. In another study, Gopal *et al*. tested escalating doses of fludarabine in addition to myeloablative RIT as a conditioning regimen for ASCT in 36 patients older than 60 years. Interestingly, post-transplant remission status included CR/CRu = 79%, PR = 6%, and 3-year PFS = 53% [39]. Deshayes *et al*. showed that intensive tandem myeloablative RIT and high-dose BEAM followed by two consecutive ASCTs in heavily pretreated patients with relapsed or refractory B-cell NHL. This regimen resulted in favorable results, with 64% (14 out of 22) CR and 23% PR, and a median OS of 101.5 months [40]. Wagner *et al*. investigated myeloablative 131I-rituximab with or without high dose chemotherapy, as conditioning regimen prior to ASCT. In median follow-up of 9.5 years, their 23 patients achieved interesting median OS and PFS of 101.5 and 47.5 months [41]. However, in another study, Berger *et al*. showed that myeloablative RIT and BEAM administered with ASCT was not associated with significantly improved survival compared with rituximab and BEAM, although the size of the RIT group was small. They compared 35 patients with MCL who underwent rituximab and BEAM, with 11 patients who received 90Y-ibritumomab tiuxetan prior to BEAM ASCT. They observed that the 5-year OS in the R-BEAM and Z-BEAM groups was 55 and 71% (p = 0.288), respectively, and the 4-year PFS was 32 and 41%, respectively (p = 0.300) [42]. Gopal *et al*. compared high dose RIT (131I-tositumomab) with high dose conventional conditioning regimen (chemotherapy plus TBI) in 125 relapsed follicular lymphoma patients. RIT group had significantly higher OS and PFS in comparison with conventional conditioning regimen (5-year OS and PFS were 67 and 48%, for RIT and 53 and 29%, for C-HDT) [43].

Based on aforementioned studies, adding high dose RIT to conventional conditioning regimens prior to ASCT provides promising and durable responses even in elderly, high risk NHL patients which may be due to its ability in eliminating residual disease. However, further studies are needed to compare advantages and disadvantages of using standard or myeloablative doses of RIT as the conditioning regimen.

## Toxicity & engraftment

The addition of standard or high dose RIT to the conditioning regimen before ASCT is well tolerated [21]. Adding RIT in comparison with not adding it to chemotherapy prior to ASCT for NHL patients caused no significant additional toxicity [29,44,45].

### Hematologic toxicities

The most common toxicity of the RIT conditioning regimen is hematologic toxicity [40]. In 90Y-ibritumomab tiuxetan and BEAM conditioning versus rituximab and BEAM conditioning prior to ASCT in MCL patients, no significant differences in hematologic toxicities or the median number of red blood cell and platelet transfusions were seen between the two groups [42]. In another comparison of rituximab and BEAM conditioning with 131I-tositumomab and BEAM conditioning for relapsed DLBCL, hematologic toxicities were not significantly different [31].

### Nonhematologic toxicities

It seems that adding RIT to conventional conditioning regimens does not add to nonhematologic toxicities either [23]. In a study comparing 90Y-ibritumomab tiuxetan and BEAM conditioning with rituximab and BEAM conditioning, no statistically significant differences in nonhematologic toxicities were observed [42]. In a randomized study of rituximab and BEAM compared with 131I-tositumomab and BEAM conditioning, mucositis score measured by the Oral Mucositis Assessment Scale was the only nonhematologic toxicity that was significantly higher in RIT and BEAM conditioning treatment group [31]. In another study comparing 90Y-ibritumomab tiuxetan and busulfan, cyclophosphamide, and etoposide (BuCyE) conditioning with BuCyE conditioning alone, there was no statistically significant difference in percentage of patients with nonhematologic toxicities [30]. Therefore, it seems that adding RIT to a conditioning regimen does not add to nonhematologic toxicities except for mucositis.

### Mortality rate

Mortality rate is another toxicity indicator reported by the studies which have added RIT to conventional conditioning regimens. In 90Y-ibritumomab tiuxetan and BEAM versus rituximab and BEAM conditioning in patients with MCL there were no early treatment-related deaths in both groups [42]. In a randomized study of rituximab and BEAM compared with 131I-tositumomab and BEAM as conditioning therapy for relapsed DLBCL, the 100-day treatment related mortality and distribution of primary causes of death were not significantly different between the two groups [31]. In another study comparing 90Y-ibritumomab tiuxetan and BuCyE conditioning with BuCyE conditioning alone, there was no statistically significant difference in percentage of patients with treatment-related mortality [30]. In matched-cohort analysis of 90Y-ibritumomab tiuxetan and BEAM versus TBI conditioning for poor-risk DLBCL, the 100-day mortality rate was 0% in the RIT group and 8.7% in the TBI group [8]. Cassaday *et al*. compared high dose 131I- tositumomab with conventional conditioning regimen prior to ASCT in 162 MCL patients. In multivariate analysis RIT conditioning was associated with a reduced risk of mortality (hazard ratio: 0.49; p = 0.01) [38].

### Secondary malignancies

The incidence of secondary malignancies is a major safety concern regarding RIT. In a meta-analysis of outcomes after 90Y-ibritumomab tiuxetan and BEAM as the conditioning regimen for SCT in DLBCL, the incidence rate of myelodysplastic syndrome (MDS) was 2.5% (95% CI: 0.7–8.5) for 98 patients in three studies [26]. In autograft with peripheral blood stem cells after high-dose 90Y-ibritumomab tiuxetan conditioning, the 5-year cumulative incidence of secondary MDS and acute myelogenous leukemia (AML) sMDS/AML was 8.29%. There was no significant difference in the cumulative incidence of sMDS/AML between the high dose-RIT patients and patients who received a high-dose sequential chemotherapy regimen followed by a chemotherapy-based myeloablative conditioning regimen (p = 0.655) [20]. In a study of 90Y-ibritumomab tiuxetan and BEAM conditioning versus rituximab and BEAM conditioning in patients with MCL, after a median follow-up of 28 months, no secondary malignancies were observed in either of the groups [42]. In a randomized study of rituximab and BEAM compared with 131I-tositumomab and BEAM conditioning for relapsed DLBCL, after a median follow-up of 25.5 months, one case of MDS was reported in each arm of the trial, and one case of AML was reported in the rituximab-BEAM arm [31]. In matched-cohort analysis of 90Y-ibritumomab tiuxetan and BEAM versus TBI conditioning for high-risk DLBCL, there was one case of MDS in the RIT group, and two cases of AML in the TBI group after median follow-up of 59.9 months [8]. Gopal *et al*. compared high dose RIT with high dose conventional conditioning regimen prior to ASCT in 125 relapsed follicular lymphoma patients. The probability of secondary MDS/AML at 8 years was 0.076 in RIT and 0.086 in conventional group, respectively [43].

### Engraftment

Some studies have investigated engraftment after RIT conditioning. In RIT and high-dose BEAM conditioning study in relapsed NHL, the median time to neutrophil engraftment was similar to patients receiving BEAM alone [23]. In a study of 90Y-ibritumomab tiuxetan and BEAM versus rituximab and BEAM conditioning prior to ASCT in patients with MCL, the median time to engraftment for neutrophils was not significantly different between the two groups [42]. In a randomized study of rituximab and BEAM compared with 131I-tositumomab and BEAM for relapsed DLBCL, engraftment was similar in the two groups, with an absolute neutrophil count of 500/l by day 28 in 93.5% of patients in the rituximab and BEAM arm, compared with 96.1% of patients in the RIT and BEAM group (p = 0.40) [31]. In another study, there was no statistically significant difference in the median time to neutrophil and platelet engraftment between the conditioning regimens of 90Y-ibritumomab tiuxetan plus BuCyE and BuCyE alone [30].

Therefore, it seems that adding standard or high dose RIT to the conventional conditioning regimen prior to ASCT does not add to hematologic toxicities, nonhematologic toxicities, mortality rate, secondary malignancy rate, and time to engraftment. However, further studies with longer follow-ups are needed.

## Discussion & conclusion

NHL is the most common hematologic malignancy. In USA in 2018, NHL is expected to be the ninth cause of death with 19,910 estimated deaths and 74,680 new cases with 5-year survival rate of 71% [46]. ORR to currently available interventions for NHL ranges widely from 9 to 97% [47–49]. Although advanced stage aggressive NHL are considered chemotherapy-responsive tumors, many patients either relapse or never achieve a remission. For these patients, a therapeutic approach based on high-dose therapy followed by ASCT is the only possibility of cure. However, up to a third of patients may never receive transplant, mostly due to progressive disease. Furthermore, relapse still remains a major concern even after transplant. Various conditioning regimens have been used before ASCT, with DFS and OS rates ranging approximately from 30 to 70%. To date, few comparative randomized trials have been performed and no regimen has demonstrated superiority to another. Furthermore, little is known about the relative toxicity and efficacy of single conditioning regimens applied in lymphomas. Advances in the conditioning regimens and supportive care have reduced transplant-related mortality in the autologous setting. However, the commonly utilized conditioning regimens have pros and cons, and new conditioning strategies are required and considered by several investigators.

Different chemotherapeutic agents have been developed as conditioning regimens prior to ASCT to promote survival of NHL patients and reduce adverse events at the same time. Recently RIT has been introduced as a novel conditioning regimen prior to ASCT in NHL patients. There are currently two different approaches to the use of RIT as conditioning regimen: standard dose of RIT (nonmyeloablative) combined with HDT; and high dose of RIT (myeloablative) with or without combination with chemotherapy agents.

In conclusion, this review indicates a promising role for RIT-based conditioning regimens for SCT (Table 1), particularly in patients who cannot tolerate HDT and/or TBI. Moreover, the addition of standard or high dose of RIT to conventional conditioning regimens significantly improves response and survival following ASCT, if compared with historical regimens such as BEAM or BEAC. Based on studies discussed in this paper, standard dose 90Y-ibritumomab tiuxetan plus BEAM significantly improves the survival of NHL patients in comparison with BEAM alone; however, there are few studies that investigated myeloablative dose of 90Y-ibritumomab as conditioning regimen or combination of 90Y-ibritumomab with other chemotherapy regimens. This improvement is also present in high-risk patients whose critical condition requires more efficient conditioning regimens.

**Table 1.** **Summary of studies using radioimmunotherapy prior to autologous stem cell transplantation.**

**No. of patients**	**Mean age (years)**	**NHL subtype**	**Regimen prior to ASCT**	**Median follow-up**	**Survival**	**No. of MDS/AML**	**Ref.**
77	18–65	FL, MZL	90Y-ibritumomab tiuxetan and BEAM	28 months	2-year EFS of 63%	1	[19]

53	64	FL, DLBCL, MZL, MCL, lymphoplasmacytic lymphoma	Myeloablative dose of 90Y-ibritumomab tiuxetan	49 months	5-year EFS of 68%	4	[20]

46	56.5	DLBCL	90Y-ibritumomab tiuxetan and BEAM	59.9 months	4-year PFS of 59.6%	1	[8]

46	53	DLBCL	TBI	59.9 months	4-year PFS of 42% (p = 0.10)	2	

63	59.5	Transformed DLBCL	90Y-ibritumomab tiuxetan and high-dose BEAM	28 months	2-year PFS of 68%	0	[21]

40	54	DLBCL	131I- tositumomab with BEAM	6 years	5-year PFS of 70%	2	[22]

23	51	FL, DLBCL, MCL	131I- tositumomab with high-dose BEAM	38 months	3-year EFS of 39%	2	[23]

22	58	Transformed FL, DLBCL	90Y-ibritumomab tiuxetan and BEAM	29 months	2-year PFS of 59%	0	[25]

21	51	Transformed FL, DLBCL	BEAM	29 months	2-year PFS of 37% (p = 0.2)	0	

19	51	FL, DLBCL, BL	90Y-ibritumomab tiuxetan and BU/CY/E	29.4 months	3-year EFS of 26.3%	0	[29]

19	54	FL, DLBCL, MZL, MCL, BL	90Y-ibritumomab tiuxetan and BU/CY/E	30.4 months	Median EFS of 12.5 months	0	[30]

19	52	FL, DLBCL, MZL, MCL, BL	BU/CY/E	30.4 months	Median EFS of 6.2 months, (p = 0.23)	0	

111	56.8	DLBCL	131I-tositumumab and BEAM	25.5 months	2-year PFS of 47.9%	1	[31]

113	58.5	DLBCL	Rituximab and BEAM	25.5 months	2-year PFS of 48.6% (p = 0.94)	2	

30	62	FL, DLBCL, MCL, MZL, lymphocytic lymphoma	3 cycles of DHAP or CHOP and high-dose 90Y-ibritumomab tiuxetan	30 months	30 months EFS of 69%	0	[33]

31	51	FL, DLBCL, MCL	High-dose 90Y-ibritumomab tiuxetan and high-dose etoposide and cyclophosphamide	22 months	2-year RFS of 78%	0	[34]

24	64	FL, DLBCL, MCL, MZL	High-dose 131I-tositumomab	2.9 years	Estimated 3-year PFS of 51%	2	[35]

44	54	FL, DLBCL, MCL	90Y ibritumomab tiuxetan and high dose BEAM	33 months	Estimated 3-year PFS of 43%	1	[36]

16	54	MCL	High-dose 131I-tositumomab and etoposide, and cyclophosphamide	19 months	Estimated 3-year PFS of 61%	0	[37]

61	57	MCL	131I-tositumomab alone or in combination with escalating doses of fludarabine or cyclophosphamide and etoposide	61 months	5-year PFS of 45%	3	[38]

101	55	MCL	BEAM, BuMelT and TBI plus cyclophosphamide with or without etoposide	61 months	5-year PFS of 49% ( p = 0.77)	3	

36	65	FL, DLBCL, MCL	Myeloablative 131I-tositumomab with escalating doses of fludarabine	3.9 years	Estimated 3-year PFS of 53%	3	[39]

23	Not reported	Majority of FL	Myeloablative 131I-rituximab and high dose BEAM followed by two consecutive ASCT	9.5 years	Median PFS of 47.5 months	3	[40]

11	57	MCL	90Y-ibritumomab tiuxetan and BEAM	43 months	4-year PFS of 41%	0	[42]

33	59	MCL	Rituximab and BEAM	28 months	4-year PFS of 32% (p = 0.30)	0	

27	46	FL	High dose 131I-tositumomab	8 years	Estimated 5-year PFS of 48%	2	[43]

98	49	FL	TBI plus chemotherapy or chemotherapy alone (including cyclophosphamide, etoposide, busulfan, melphalan, thiotepa, carmustine)	7 years	Estimated 5-year PFS of 29% ( p = 0.03)	6	

16	61	Transformed/aggressive FL, DLBCL, MCL, MALT	131I rituximab with BEAM	44 months	3-year EFS of 64%	0	[44]

AML/MDS: Acute myeloid leukemia/myelodysplastic syndrome; ASCT: Autologous stem cell transplantation; BEAM: Etoposide, rabinoside, cytarabine and melphalan; BL: Burkitt lymphoma; BU/CY/E: Busulfan, cyclophosphamide and etoposide; BuMelT: Busulfan, melphalan and thiotepa; CHOP: Cyclophosphamide, doxorubicin hydrochloride, vincristine and prednisolone; DHAP: Dexamethasone, high-dose cytarabine and cisplatin; DLBCL: Diffused large B-cell lymphoma; EFS: Event-free survival; FL: Follicular lymphoma; MCL: Mantel cell lymphoma; MZL: Marginal zone lymphoma; NHL: Non-Hodgkin lymphoma; PFS: Progression-free survival; RFS: Relapse-free survival; TBI: Total body irradiation.

Last but not least, it seems that the enhanced efficacy is not accompanied with any addition to toxicity or delayed engraftment, making this option particularly attractive for elderly patients. Comparative studies showed that adding high dose 90Y-ibritumomab to conditioning regimen would not add to hematologic, nonhematologic toxicities and time to engraftment; even it could reduce the secondary malignancies and mortality rate in comparison with conventional TBI-containing conditioning regimens. However, results are not unequivocal and therefore the addition of RIT to HDT cannot be recommended outside from clinical trials.

## Future perspective

RIT as a novel therapeutic modality has been used in NHL patients in the past decade. As it was discussed in this paper, using RIT as conditioning regimen prior to ASCT could provide a favorable and durable response in NHL patients; however, there are still ambiguities in this field which call for further future studies. Randomized clinical trials are needed to compare the exact potential superiorities of standard dose of RIT to rituximab as a conditioning regimen and to clarify which chemotherapy regimen is the best for combination with RIT as a conditioning regimen. Moreover, it is still not clear which NHL patients are suitable for applying a myeloablative dose of RIT. High risk NHL patients are more prone to relapse, thus they need more potent conditioning regimens including high dose RIT; however, myeloablative toxicities would be an important concern for this group of patients. Currently there are few randomized trials with long term follow-up in order to clarify the exact toxicities of high dose RIT. A promising future could be pictured for RIT as a conditioning regimen prior to ASCT, if further studies focus on clarifying these questions.
